# The Medicinal Potential of Mesenchymal Stem/Stromal Cells in Immuno- and Cancer Therapy

**DOI:** 10.3390/biom13081171

**Published:** 2023-07-27

**Authors:** Mehdi Najar, Hassan Fahmi, Makram Merimi

**Affiliations:** 1 Faculty of Medicine, Université Libre de Bruxelles, 1070 Brussels, Belgium; 2 Osteoarthritis Research Unit, Department of Medicine, University of Montreal Hospital Research Center (CRCHUM), Montreal, QC H2X 0A9, Canada; 3 LBBES Laboratory, Genetics and Immune Cell Therapy Unit, Faculty of Sciences, University Mohammed Premier, Oujda 60000, Morocco

Cancer is a highly lethal disease that causes millions of deaths worldwide, thus representing a major public health challenge. Conventional therapeutic approaches, such as surgery, chemotherapy, and radiation therapy, have limitations in terms of their targeting and specificity. Additionally, the development of molecular and immunological aberrations associated with targeted therapies often leads to cancer therapy resistance and tumor recurrence. In recent years, cellular and acellular treatments have emerged as new categories that have transformed the landscape of cancer treatment [1]. Actually, it is clearly admitted that non-hematopoietic stromal cells harboring immune functions, such as mesenchymal stem stromal cells (MSCs), are critical for tissue homeostasis. MSCs have, for a long time, been recognized as pivotal contributors to the set up and maintenance of the hematopoietic niche. MSCs are a cellular therapeutic product whose potential in managing several diseases and disorders is being investigated. These cells are involved in immunomodulation, resolution of inflammation, and regeneration of injured/damaged tissues [2].

They are also reported to be a component of the tumor microenvironment (TME) due to their showing homing capacity towards tumor sites [3]. MSCs may therefore interact with cancer and immune cells and thus modulate their fate and functions. The conflicting roles of MSCs in tumor inhibition and tumor growth impede their adaptation for anticancer therapies. MSCs are a multipotent adult cell population present within several niches participating in the maintenance and regeneration of multiple tissues. MSCs were first isolated from bone marrow (BM) but have subsequently been isolated from umbilical cord and adipose tissues, among others [4]. MSCs can be readily isolated from these sources and expanded ex vivo without any apparent modification in phenotype or loss of function. In vitro, MSCs are able to differentiate along multiple lineages including osteoblasts, chondrocytes, adipocytes, myocytes, neural precursors, and possibly other cell types. However, there are several conflicting opinions in the literature regarding the contribution of the multilineage potential in the therapeutic functions of MSCs. Furthermore, MSCs have been reported to modulate the inflammatory and immune responses by regulating different immune cell populations. The absence of costimulatory molecules allows MSCs to be considered inherently poorly immunogenic. However, recent preclinical studies have described the generation of alloantibodies and the immune rejection of MSCs. This has led to an increasing number of clinical trials evaluating the immunological profile of patients after treatment with MSCs [5]. The immunomodulation–immunogenicity balance of MSC is differentially affected by the immune cell response depending on inflammatory licensing and MHC compatibility. Indeed, the immunomodulatory effects of MSCs are not constitutive but induced by different signals present in their surroundings [6]. Depending on the inflammatory stimuli, MSCs show high functional plasticity with the capacity either to inhibit the immune response or to enhance it. The immunological response to inflammation is also specific to each MSC type and involves distinct modifications within their ncRnome, transcriptome, and secretome. These changes in the MSC microenvironment can differentially modulate the immune response and therefore, tumor progression as well as drug-resistance. Thus, guiding MSCs toward a specific immunological profile and function is a challenge in order to boost anticancer properties while eliminating tumor-favoring effects. Such polarization may be associated with the pro- and anti-cancer effects of MSC, probably due to the distinct expression of bioactive molecules [7]. The use of MSCs represents a promising approach to cancer therapy, particularly in the field of oncohematology, owing to their tropism towards tumors. The emerging role of MSCs in creating a protective and immune-tolerant microenvironment may support the survival of tumor cells and affect the response to therapies. In this light, it has been proposed that the failure of current treatments to efficiently override the stroma-mediated protection of tumor cells accounts for the high rate of relapse in cancer, at least in part. Research on the therapeutic applications of MSCs must give consideration to the fact that they exert both antitumorigenic and protumorigenic effects on hematologic malignancies. Thus, the ex vivo pre-conditioning and engineering of MSC, and therefore, the understanding of their plasticity, is a valuable strategy to enhance anti-cancer immune responses [8]. As a cell-free therapeutic tool, extracellular vesicles (EVs) derived from MSCs have been identified as a valuable therapeutic approach. The efficacy of EVs is due to their cargo—bioactive molecules. Indeed, MSCs are likely to exert their effects by releasing a variety of biologically active molecules such as mRNAs, non-coding RNAs (ncRNAs), proteins, growth factors, chemokines, and cytokines, either as soluble proteins or enclosed in EVs. EVs are attracting significant attention in the world of medicine, due to their ability to mimic most of the therapeutic effects of MSCs [9]. A growing number of studies suggest that MSC-derived EVs may mimic the pro- and anti-tumor effects of MSCs. Genetic engineering can be utilized to equip EVs with a specific tumor tropism and therefore, may constitute an alternative cancer treatment. New approaches that can repolarize and/or promote the anti-cancer immune response by using MSCs or their EVs should be investigated. Importantly, the pre-conditioning or engineering of MSCs may strengthen the anti-cancer immune response by generating pro-inflammatory MSCs or EVs with anti-tumor effects in parallel, to increase the cytotoxic functions of immune effector cells. Thus, characterization and modulation of the immunological features of MSCs and their EVs in cancer patients may serve as a basis for enhancing the anti-tumor immune response and, ultimately, for finding a cure. Profiling MSCs or their EVs may lead to the identification of a suitable candidate with potential regarding tumorogenesis, or in new treatment strategies. Much remains to be determined with regard to the immunological interplay between the anti-tumor immune response and MSCs. This aim of this Special Issue of Biomolecules is to present and discuss new perspectives for targeted cancer therapies based on the use of MSCs or their EVs. Contributions that enhance our understanding of novel and innovative strategies for cancer management and immune modulation are encouraged.
